# The role of high-sensitivity C-reactive protein serum levels in the prognosis for patients with stroke: a meta-analysis

**DOI:** 10.3389/fneur.2023.1199814

**Published:** 2023-06-05

**Authors:** Liuting Chen, Min Wang, Chanrui Yang, Yefei Wang, Bonan Hou

**Affiliations:** ^1^The Second Clinical Medical College, Zhejiang Chinese Medical University, Zhejiang, Hangzhou, China; ^2^Department of Neurology, The Second Affiliated Hospital of Zhejiang Chinese Medical University, Zhejiang, Hangzhou, China

**Keywords:** high-sensitivity C-reactive protein, prognosis, meta-analysis, ischemic stroke, hemorrhagic stroke

## Abstract

**Background:**

The impact of high-sensitivity C-reactive protein (hs-CRP) as a biomarker of inflammation on the prognosis of stroke patients remains controversial, this study was conducted to evaluate the prognostic value of hs-CRP levels for patients with stroke.

**Methods:**

PubMed, Web of Science, Embase, and Cochrane Library databases were searched from inception to October 28, 2022. Outcome measures were all-cause mortality, recurrent stroke, and poor prognosis. The relationship between the highest versus lowest levels of hs-CRP or per unit increment and outcomes as measured by risk ratio (RR) and corresponding 95% confidence intervals (CI).

**Results:**

A total of 39 articles were eligible for meta-analysis. High hs-CRP levels at admission were associated with mortality among patients with acute ischemic stroke (AIS) [RR = 3.84, 95% CI (2.41 ~ 6.111); *p* < 0.001], risk of recurrent stroke [RR = 1.88, 95%CI (1.41 ~ 2.52); *p* < 0.001], and poor prognosis [RR = 1.77, 95% CI (1.59 ~ 1.97); *p* < 0.001]. The risk ratios for the association of per unit increase in hs-CRP levels with mortality, risk of recurrent stroke, and poor prognosis were as follows, respectively: 1.42 [95% CI (1.19–1.69); *p* < 0.001], 1.03 [95% CI (1.01–1.04); *p* = 0.003], and 1.27 [95% CI (1.10–1.47); *p* = 0.001]. For hemorrhagic stroke (HS), the risk ratios (RR) for the highest versus the lowest (reference) category of hsCRP or per unit increment to all-cause mortality were 4.36 [95% CI (1.38–13.73); *p* = 0.012] and 1.03 [95% CI (0.98–1.08); *p* = 0.238].

**Conclusion:**

Hs-CRP levels are strongly associated with mortality, risk of stroke recurrence and poor prognosis in stroke patients. Therefore, hs-CRP levels may contribute to the prognosis prediction of these patients.

## 1. Introduction

Stroke is the second leading cause of death following ischemic heart disease, accounting for 11.6% of total deaths (1). Particularly, ischemic stroke (IS) makes up around 87% of stroke cases (2). In China, the burden of stroke data in 2020 revealed that the number of deaths related to stroke reached a staggering 2.3 million (3). The wide application of modern secondary prevention therapy may be counterbalanced by a high risk of further vascular events in stroke survivors (4) which has become an increasing burden on public health worldwide. Therefore, it is of great significance to explore key factors affecting the prognosis of patients with stroke to formulate appropriate treatment regimens and optimize healthcare for these patients.

The pathogenesis of stroke mainly includes oxidative stress and inflammation. Inflammatory factors can not only induce cell death responsible for functional injury (5), but also underlie the development of atherosclerosis by regulating macrophages, cytokines, and leukocyte adhesion molecules to induce endothelial dysfunction, plaque formation and rupture, platelet aggregation, and thrombosis (6, 7). Therefore, some inflammatory cytokines are investigated as predictors of functional outcomes after stroke (8).

High-sensitivity C-reactive protein (hs-CRP), which is synthesized and secreted by liver cells, is considered a non-specific biomarker of inflammation (9). Previous meta-analyses have shown that hs-CRP can be used to predict the prognosis of patients with COVID-19 (10), type 2 diabetes (11), or coronary artery disease (12), and they also indicate that the level of hs-CRP is an independent risk factor for different types of stroke (13). However, its use as a biomarker to predict patient prognosis after stroke remains controversial. Zeng et al. (14) showed that a high level of hs-CRP level was an independent predictor of adverse clinical outcomes in patients with stroke. Zhang et al. (15) pointed out that the risk of recurrent stroke in patients with IS increased by 22% with a per unit increase in the level of hs-CRP. However, some studies suggest that elevated hs-CRP levels do not seem to independently affect the outcome in patients with stroke (16).

Furthermore, previous meta-analyses have only focused on the impact of hs-CRP on the mortality (17) or stroke recurrence (18) prognosis of ischemic stroke patients, without considering the overall mortality prognosis of hemorrhagic stroke patients, and lacking analysis of poor outcomes based on mRS scores. Therefore, the present study aims to comprehensively evaluate the prognostic value of hs-CRP levels for patients with stroke by performing a meta-analysis to investigate the correlation between hs-CRP levels with recurrent stroke, mortality, and poor prognosis in patients with ischemic or hemorrhagic stroke.

## 2. Methods

Databases PubMed, Web of Science, Embase, and Cochrane Library were searched from inception to October 28, 2022. The following search terms were used: “C-reactive protein or high-sensitivity CRP or hs-CRP or hsCRP” and “stroke or intracerebral hemorrhage or TIA or CVA or Brain Vascular Accident or Cerebrovascular Accident or Apoplexy” and “observational or cohort or case–control or cross-sectional or follow up or prospective or retrospective.” The literature search was independently done by two researchers, and discussion among them was undertaken to settle disagreements if any. Reference lists of relevant studies were retrieved to conduct a sensitive search. The literature search strategy is presented in Supplemental methods. This study has been registered in PROSPERO (CRD42023389330).

### 2.1. Eligibility criteria

Inclusion criteria: (1) Adult patients diagnosed with stroke (ischemic stroke or hemorrhagic stroke) participated in studies, (2) baseline hs-CRP levels were measured after symptom onset or at the time of admission (before treated), (3) patients with IS were followed up for no less than 3 months, (4) risk ratio (RR) or odds ratio (OR) with a 95% confidence interval (CI) for prognostic indicators were provided in studies, or sufficient data were available to calculate RR or OR with 95% CI, and (5) observational studies were included in the meta-analysis.

Exclusion criteria: (1) conference abstracts, case reports, letters, reviews, and animal experiments were excluded; (2) studies that were not published in English were excluded; (3) duplicate publications or articles whose full texts were not available were excluded; (4) studies investigating endpoints other than death, poor prognosis (as assessed by the mRS Scale), or recurrent stroke were excluded.

### 2.2. Data collection and quality assessment

Information extracted from eligible studies included first author, year of publication, country, study type, sample size, age, sex, type of stroke, cut-off value of hs-CRP, adjusted factors, duration of follow-up, and outcome measures. The quality of included studies was evaluated using the Newcastle-Ottawa Scale (NOS) within three categories: selection, comparability, and exposure. A study can be scored on a scale of 0 to 9, with a score of 6 or higher indicating high quality.

### 2.3. Statistical analysis

All statistical analyses were performed using STATA software (version 15.0, STATA, College Station, TX, United States). Dichotomous and continuous variables were both expressed as RR with corresponding 95% CI to evaluate the correlation of hs-CRP levels with the prognosis in stroke patients. The Q test and *I*^2^ statistics were used to assess the heterogeneity across the included studies. If significant heterogeneity existed between studies (*I*^2^ ≥ 50%, *p* < 0.10), a random-effects model was applied; otherwise, a fixed-effects model was used. Subgroup analysis was conducted to find the source of heterogeneity. Sensitivity analysis was used to assess the stability of pooled results. Begg’s test and Egger’s test were done to evaluate publication bias, with a value of *p* <0.05 indicating statistical significance.

## 3. Results

### 3.1. Literature search and screening results

**Figure 1:**
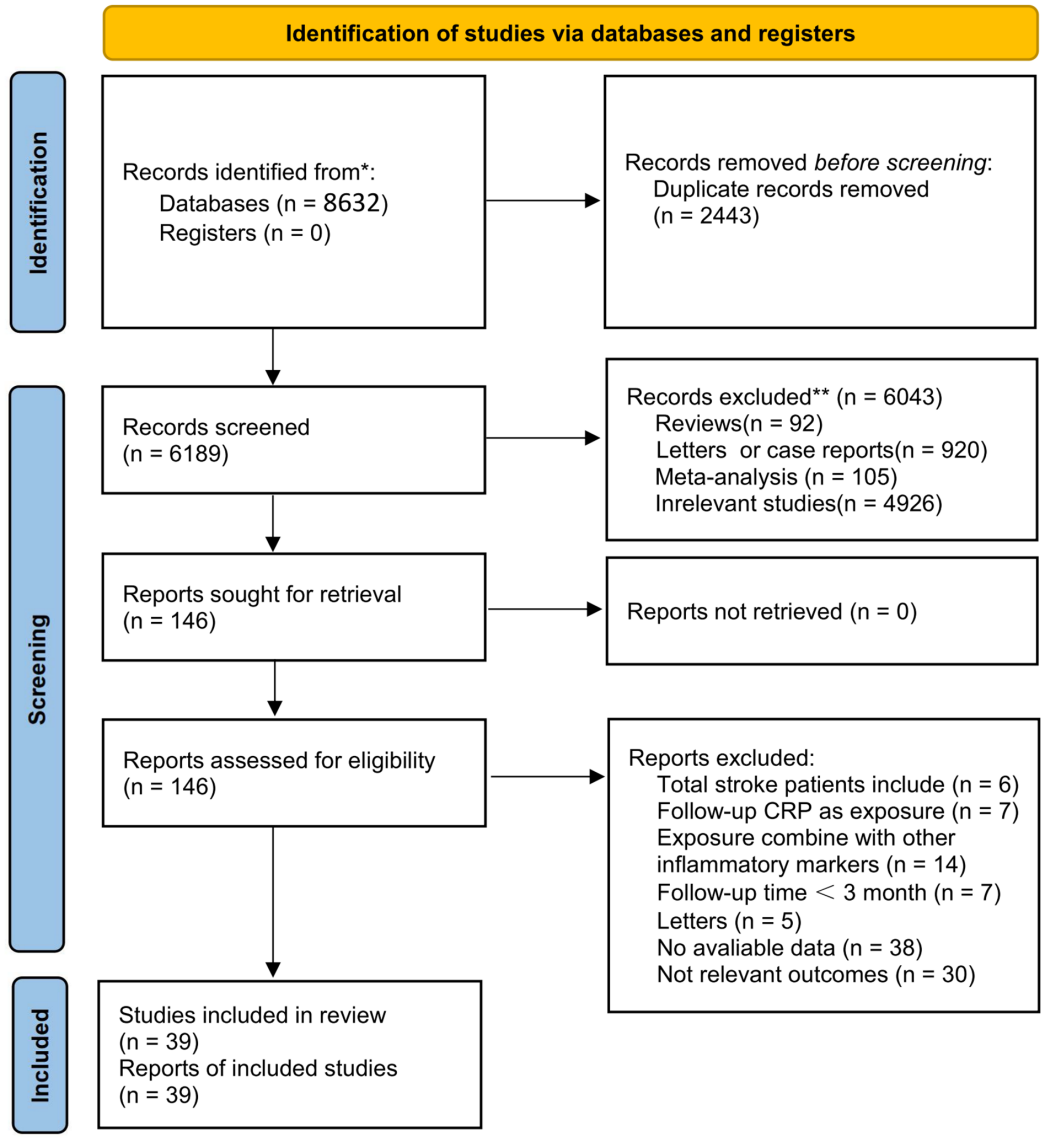
Flow diagram of the study selection process.

A literature search in databases yielded 8,632 articles in total. Among them, 2,443 articles were removed for duplicate publication. Screening of titles and abstracts excluded irrelevant articles such as meta-analyses, reviews, and animal experiments. Then, 146 full-text articles were reviewed. Finally, 39 articles met the criteria for inclusion in the meta-analysis (Figure 1).

### 3.2. Basic characteristics of included studies

Table 1 presents the basic characteristics of included studies. Among 39 studies, 23, 10, and 15 articles, respectively, reported mortality, recurrent stroke, and poor prognosis among patients with stroke. Participants with IS were included in 33 studies (26 prospective cohort studies and 7 retrospective cohort studies) and those with HS were included in 6 studies (4 prospective cohort studies and 2 retrospective cohort studies). The sample size in studies investigating IS ranged from 89 to 9,438, amounting to 41,175 participants in total (27,140 men and 14,035 women), with the duration of follow-up ranging from 3 months to 7.4 years. Among these studies, some (*n* = 2) stratified IS into large artery atherosclerosis (LAA), cardiogenic embolism (CE), small-artery occlusion (SAO), etc. However, some studies only investigated one specific subtype of ischemic stroke: LAA (*n* = 4), SAO (*n* = 2), and CE (*n* = 1). The subtype of HS that was more frequently investigated by the included studies was intracerebral hemorrhage (ICH), with the sample size ranging from 91 to 329. As for NOS scores, included studies were awarded from 6 to 9, indicating that they had moderate or high quality.

**Table 1:** Baseline characteristics of studies included in meta-analysis.

Author	Year	Country	Study design	Study size	Age	Gender (M/F)	Stroke type	hsCRP cutoff	Adjusted factor	Follow up	Outcome	Overall NOS
Ischemic stroke												
Coveney et al. (19)	2022	Ireland	PC	238	58–83	144/94	IS/TIA	NA	ABCD2 score and DWI-positivity	1 year	3-month stroke recurrence	7
Wang et al. (16)	2022	China	PC	9,438	63 (54–70)	6732/2706	AIS/TIA	Q1: <0.82 mg/L; Q2: 0.82–1.77 mg/L; Q3: 1.77–4.71 mg/L;Q4: ≥4.71 mg/L T1: <1; T2:1–3; T3:≥3	age, sex, body mass index, current smoking, index event, medical histories of atrial fibrillation, coronary heart disease, stroke, diabetes, hypertension and hypercholesterolemia, baseline National Institutes of Health Stroke Scale score and baseline leukocyte count, rt-PA treatment, pre-mRS score, and the Org 10,172 test in the Treatment of Acute Stroke (TOAST) classification.	1 year	1-year stroke recurrence	7
Pu et al. (20)	2022	China	RC	119	68 (58, 72)	75/44	LAA/CE/SAO	NA	age, sex, smoking, alcohol consumption, NIHSS scores, TOAST subtype and hypertension at baseline.	3 months	month poor outcome (mRS 3–6)	7
Gu et al. (21)	2022	China	PC	7,603	62.3 ± 11.3	5211/2392	AIS	Q1: <0.81 mg/L; Q2: 0.81–1.73 mg/L; Q3: 1.73–4.38 mg/L;Q4: >4.38 mg/L	demographics (age, sex, body mass index), the National Institutes of Health Stroke Scale score at admission, smoking status, systolic blood pres. sure, diastolic blood pressure, medical history (prior stroke/ transient ischemic attack, hypertension, diabetes mellitus, dyslipidemia, prior coronary heart dis ease / myocardial infarction, atrial fib/flutter), and image data (infarction pattern, infarction location) and etiology classification	3 months	3-month stroke recurrence	8
Zhang et al. (22)	2021	China	RC	3,013	72.9 (12.8)	1801/1212	SAO/CE/LAA	NA	risk factors and TOAST subtype	3 months	3-month mortality 3-month poor outcome (mRS 3–6)	7
Zeng et al. (13)	2021	UK	PC	200	65.39	134/66	LAA	T1:<1.68; T2:1.70–5.46;T3:>5.50	gender, age, smoking history, drink ing history, history of dyslipidemia, history of diabetes, lipid levels, and blood glucose levels	1 year	poor outcome(mRS 2–6) at 3, 6, and 12 months	8
Wu et al. (23)	2021	China	PC	1772	70.60 (7.54)	1094/678	LAA	NA	NA	3 years	All-cauese mortality at 3, 12, and 36 months Stroke recurrence at 3, 12, and 36 months	6
Wu et al. (24)	2021	China	PC	1,214	61 (52, 68)	869/345	IS	NA	NA	23 months (median)	Stroke Recurrence	8
Wang et al. (25)	2020	China	PC	362	63.2 (12.7)	249/113	AIS	8.255 mg/L	NA	15 months (median)	Long-term mortality Long-term poor outcome(mRS 3–6)	7
Ma et al. (26)	2020	China	PC	288	59.3 ± 7.4	203/85	IS	NA	Age，Male，BMI，Hypertension，Diabetes，Smoking history，Drinking history，Family history of ischemic stroke，TC，TG，HDL-C，LDL-C，Lp(a)，Apo A/Apo B，NLR，PLR，TNF-a，IL-6	3 months	3-month all-cauese mortality	7
Kim et al. (17)	2020	Korea	RC	404	48–92	216/188	LVO	T1: <1; T2:1–3; T3:> = 3	age, sex, hypertension, diabetic mellitus, body mass index, initial National Institutes of Health Stroke Scale score, erythrocyte sedimentation rate, successful recanalization, and procedure time	3 months	3-month all-cauese mortality 3-month poor outcome (mRS 3–6)	7
Continued												
Author	year	Country	Study design	Study size	Age	Gender (M/F)	Stroke type	hsCRP cutoff	Adjusted factor	Follow up	Outcome	Overall NOS
Huţanu et al. (27)	2018	Romania	PC	89	71.9 ± 10	41/48	IS	T1: ≤2.86; T2: 2.86–9.48; T3: >9.48	age, gender and stroke severity (initial NIHSS scores was dichotomized in NIHSS>7 and NIHSS≤7 points)，dyslipidemia, atrial fibrillation and history of stroke	3 months	3-month poor outcome (mRS 3–6)	7
Zhang et al. (14)	2017	China	PC	286	63 (53–74)	150/136	AIS	Q1:<0.22 mg/dL;Q2:0.22–0.42 mg/dL;Q3:0.43–1.09 mg/dL;Q4:>1.09 mg/dL	age, sex, BMI, stroke syndrome, stroke etiology, the NIHSS score,infarct volume, vascular risk factors, prestroke therapy,acute treatment, and serum levels of FBG and HCY	1 year	1-year stroke recurrence	8
Ye et al. (28)	2017	China	PC	625	60(51–68)	458/167	LAA	2.40 mg/L	age and sex，hypertension, dyslipidemia, diabetes, smoking, and the NIHSS score at baseline	1 year	year poor outcome (mRS 3–6)	7
Hou et al. (29)	2017	China	PC	1,299	63.22 ± 11.27	864/435	LAA	3.215 mg/L	age and NIHSS score	3 months	3-month poor outcome (mRS 3–5)	8
				453	62.13 ± 11.38	308/145	SAO	1.72 mg/L	NIHSS score			
Gao et al. (30)	2017	China	RC	494	60.33 ± 11.19	336/158	SAO	Q1:<0.67, Q2:0.67 ~ 1.46, Q3:1.46 ~ 3.46, Q4:≥ 3.46 mg/L	hypertension and NIHSS scores	3 months	3-month poor outcome (mRS 3–5)	9
Li et al. (31)	2016	China	PC	3,044	62 (55–71)	2027/1017	AIS/TIA	Q1: <0.8 mg/L; Q2: 0.8–1.7 mg/L; Q3: 1.7–4.2 mg/L; Q4: >4.2 mg/L T1: <1; T2:1–3; T3:>3	reccurance:age, BMI, sex, medical histories of myocardial infarction, hypertension and diabetes mellitus, baseline NIHSS score, baseline leukocyte count, randomized treatment of aspirin monotherapy or dual antiplatelet therapy, and use of antihypertension agents, lipid-lowering agents, and hypoglycemic agents during follow-up poor outcome:age, sex, medical histories of hypertension, diabetes mellitus and ischemic stroke, baseline NIHSS score, baseline mRS score, baseline leukocyte count, qualifying event, randomized treatment of aspirin or dual antiplatelet therapy, and use of hypoglycemic agents and anti-hypertension agents during 90 days follow-up period.	1 year	3-month and 1-year stroke recurrence 3-month poor outcome (mRS 2–6)	7
Wang et al. (32)	2016	China	PC	376	69 (63–79)	206/170	AIS	NA	age, NIHSS, other predictors, and vascular risk factors	1 year	1-year all-cauese mortality 1-year poor outcome (mRS 3–6)	7
Matsuo et al. (33)	2016	Japan	PC	3,653	70.8 ± 12.2	2323/1330	AIS	Q1:⩽0.50 mg/L;Q2:0.50–1.25 mg/L;Q3:1.25–4.70 mg/L;Q4:˃ 4.70 mg/L	age, sex, baseline National Institutes of Health Stroke Scale score, and stroke subtypes，hypertension, dyslipidemia, diabetes mellitus, atrial fibrillation, smoking, drinking, chronic kidney disease, body mass index, intravenous thrombolytic therapy and endovascular therapy, and acute infections	3 months	3-month poor outcome (mRS 3–6)	9
Bakhshayesh-Eghbali et al. (34)	2016	Iran	PC	102	69.471 ± 12.125	43/59	AIS	NA	chronic diseases	3 months	3-month poor outcome (mRS 4–6)	6
Li et al. (35)	2015	China	PC	374	69 (63–79)	206/168	IS	NA	age, sex, smoking, glucose, HCY,NIHSS, TPA-T, infarct volume and TACS	1 year	1-year all-cauese mortality	9
Karlinski et al. (15)	2014	Poland	RC	341	61–81	170/171	AIS	5 ng/mL	Age, baseline NIHSS, DM, CHF, lack of prestroke disability, recent infection and prestroke statins use	3 months	3-month all-cauese mortality	7
Elkind et al. (36)	2014	USA	PC	1,244	63.3 ± 10.8	789/455	lacunar stroke	Q1:<0.93 mg/L;Q2:0.93–2.16 mg/L;Q3:2.16–4.86 mg/L;Q4:≥ 4.86 mg/L	demographics (age, sex, race, and region), comorbidities (hypertension, smoking, history of ischemic stroke, diabetes mellitus, body mass index, and low density and high-density lipoprotein), and statin use at baseline	3 years	3-year stroke recurrence	6
Continued												
Author	year	Country	Study design	Study size	Age	Gender (M/F)	Stroke type	hsCRP cutoff	Adjusted factor	Follow up	Outcome	Overall NOS
Tu et al. (37)	2013	China	PC	189	66 (58–75)	117/72	AIS	NA	age and the NIHSS	3 months	3-month mortality 3-month poor outcome (mRS 3–6)	8
Kuwashiro et al. (38)	2013	Japan	PC	425	76 ± 11	234/191	CE	NA	NA	1 year	1-year recurrent stroke	7
Huang et al. (18)	2012	USA	PC	741	60.9 ± 13.3	555/186	IS	3 mg/L	age, gender, National Institutes of Health Stroke Scale score (NIHSS), glucose level at admission, history of hypertension, coronary heart disease, and fasting glucose at admission	3 months	3-month all-cauese mortality	9
Corso et al. (39)	2010	Italy	PC	462	74.0(72.8–75.1)	224/238	IS	9 mg/L	NA	2.27 years (median)	Long-term all-cauese mortality	7
den Hertog et al. (40)	2009	Netherlands	PC	561	69.7	336/225	AIS	7 mg/L	age, sex, NIHSS score, cigarette smoking, diabetes mellitus, hypertension, statin use, and stroke subtype	3 months	3-month mortality 3-month poor outcome (mRS 3–6)	8
Shantikumar et al. (41)	2009	UK	RC	394	58–81	184/210	IS	Q1:<2.48 mg/L;Q2:2.48–6.62 mg/L;Q3:6.63–22.33 mg/L;Q4:≥ 22.3 mg/L	age, atrial fibrillation, previous stroke/TIA, and stroke subtype	7.4 years	Long-term all-cauese mortality	6
Montaner et al. (42)	2006	Spain	PC	143	70.65	73/70	IS	0.77 mg/L	Stroke severity	3 months	3-month all-cauese mortality	8
Winbeck et al. (43)	2002	Germany	PC	127	65(63–68)	74/ 53	IS	0.86 mg/dL	age, Barthel Index score at admission, and incidence of IHD, hypercholesterolemia,diabetes mellitus, and hypertension	1 year	1-year all-cauese mortality	9
Di Napoli et al. (44)	2001	Italy	PC	128	73.10 ± 9.17	53/75	IS	1.5 mg/dL	NA	1 year	1-year all-cauese mortality 1-year recurrent stroke	7
Muir et al. (45)	1999	Scotland	PC	228	67.0 ± 13.1	124/104	IS	NA	Age, NIHSS, TC, histories of previous MI,stroke, and cigarette smoking.	2.63 years	Long-term all-cauese mortality	8
Hemorrhagic stroke												
Sagar et al. (46)	2021	India	PC	250	54.9 ± 12.8	162/88	ICH	NA	Age, ICH volume, IVH, GCS at admission, HCY, CRP, MMP9, SELE, & SELP	3 months	3-month mortality	8
Bender et al. (47)	2021	Germany	RC	329	67.4 (13.6)	152/177	ICH	NA	NA	at discharge	intrahospital mortality	7
Elhechmi et al. (48)	2017	Tunisia	RC	91	64.35(61.54–67.17)	56/35	sICH	30 mg/L	ICH score	1 month	1-month all-cauese mortality	8
Hansen et al. (49)	2016	USA	PC	198	NA	NA	ICH	5 mg/L	NA	1 month	1-month all-cauese mortality	8
Di Napoli et al. (50)	2012	Italy	PC	223	67.4 ± 11.8	132/91	sICH	NA	demographic data (age and sex), risk factors (arterial hypertension, diabetes mellitus, alcohol abuse,hypercholesterolemia),markers of spontaneous intracerebral hemorrhage severity (Hemphill’s original ICH score, Glasgow Coma Scale score), neuroradiologic findings (ICH volume, intraventricular extension, hydrocephalus, midline shift), acute phase biomarkers (blood glucose and white blood cells at admission), and therapy	1 month	1-month all-cauese mortality	8
Di Napoli et al. (51)	2011	Italy	PC	210	67.3 (11.5)	122/88	sICH	Q1:<0.40 mg/L;Q2:0.40–7.9 mg/L; Q3:8.0–12.0 mg/L;Q4:>12 mg/L	time of blood sample delay and intracerebral hemorrhage (ICH) score variables (Glasgow Coma scale score, ICH volume, intraventricular hemorrhage, infratentorial origin of spontaneous ICH, and age,neuroradiological findings (midline shift and the presence of hydrocephalus), surgery)	1 month	1-month all-cauese mortality	8

### 3.3. Prognosis of patients with ischemic stroke

#### 3.3.1. Relationship between hs-CRP levels and all-cause mortality

**Figure 2:**
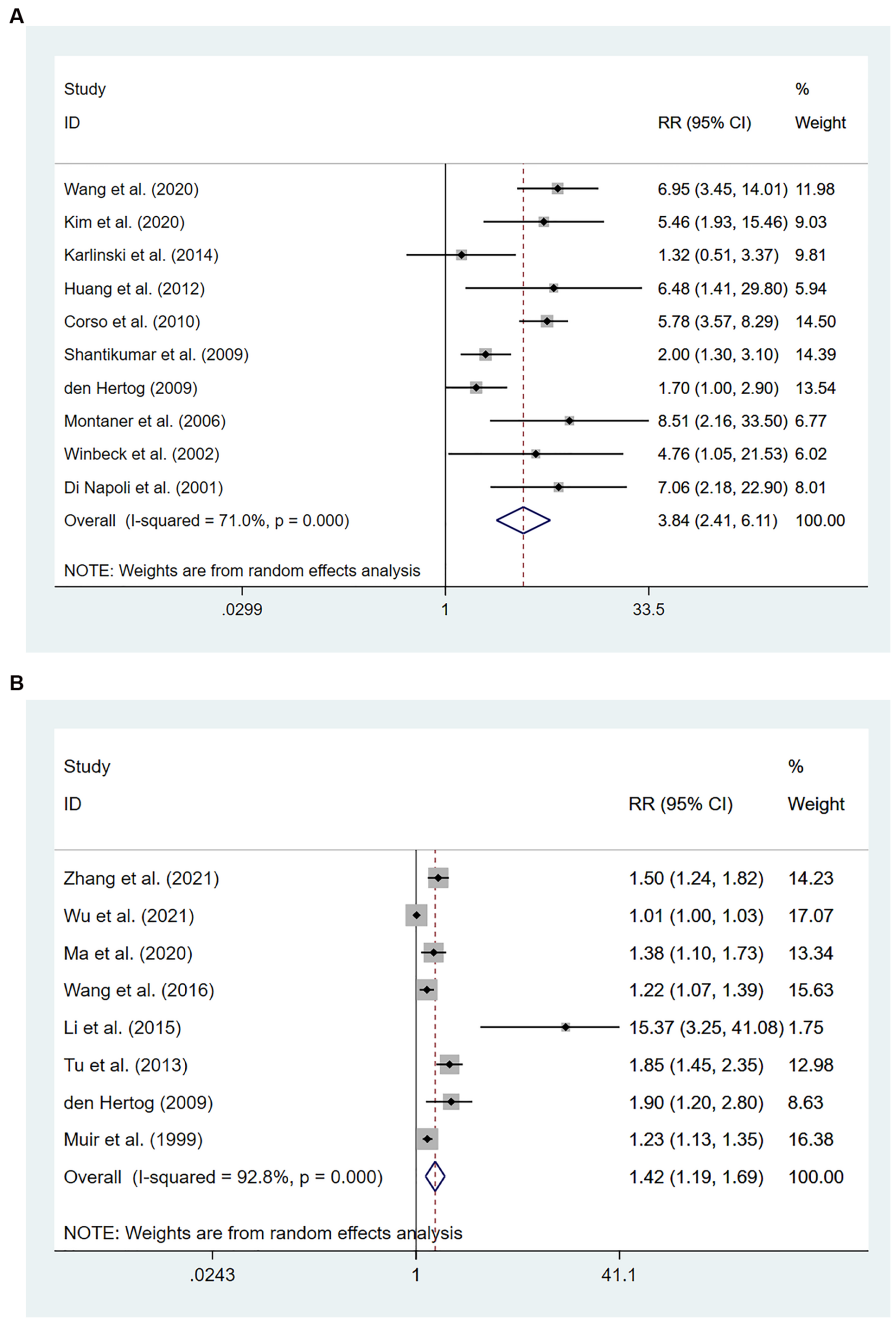
Forest plots showing risk ratios with 95% confidence intervals of all-cause mortality in ischemic stroke patients **(A)** the highest versus the lowest C-reactive protein level category; **(B)** per 1-SD rise in loge-hsCRP level.

Ten studies (16, 22, 23, 26, 39–44) involving 3,663 patients in total reported an association between high levels of hs-CRP and mortality. There was significant heterogeneity among studies (*I^2^* = 71.0%; *p* < 0.01), and thus a random-effects model was utilized. Meta-analysis showed that the risk of death increased to 384% among patients who had high hs-CRP levels upon admission, compared with those with low hs-CRP levels [RR = 3.84, 95% CI (2.41 ~ 6.111); *p* < 0.001; Figure 2A].

Eight studies (21, 31, 32, 35–37, 44, 45) with 6,801 patients in total revealed an association between per unit increase in hs-CRP levels and mortality. Heterogeneity among studies was significant (*I*^2^ = 92.8%; *p* < 0.01), and thus a random-effects model was utilized. Meta-analysis showed that per unit increase in the level of hs-CRP was associated with an increased risk of death in patients with IS [RR = 1.42, 95% CI (1.19 ~ 1.69); *p* < 0.001; Figure 2B].

#### 3.3.2. Relationship between hs-CRP levels and recurrent stroke

**Figure 3:**
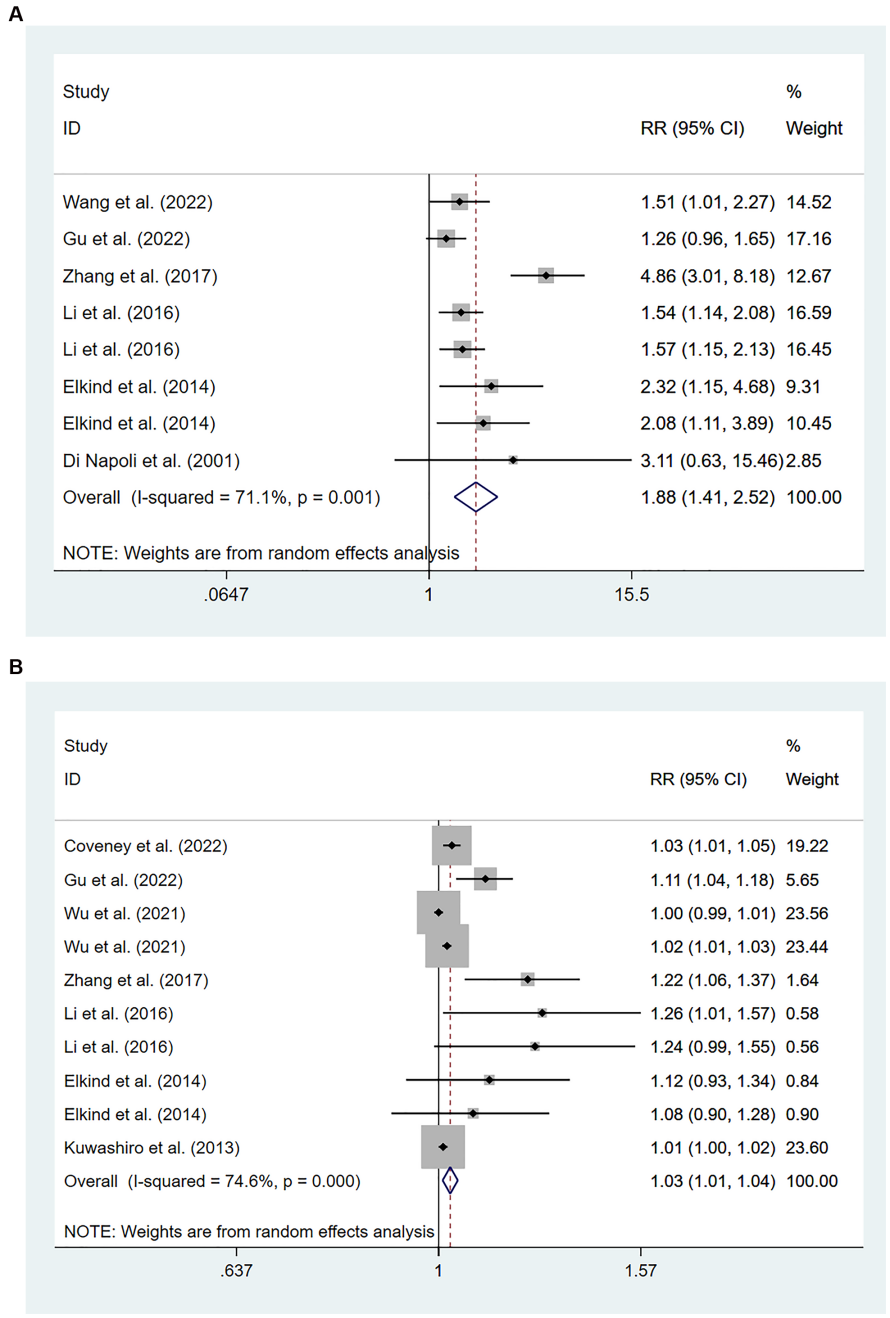
Forest plots showing risk ratios with 95% confidence intervals of stroke recurrence in ischemic stroke patients **(A)** the highest versus the lowest C-reactive protein level category; **(B)** per 1-SD rise in loge-hsCRP level.

Six studies (15, 19, 24, 26, 38, 39) involving 201,743 patients reported an association between high levels of hs-CRP and the risk of recurrent stroke. There was significant heterogeneity among studies of interest (*I*^2^ = 71.1%; *p* < 0.01), and thus a random-effects model was used for the meta-analysis. The risk of recurrent stroke in patients with high hs-CRP levels upon admission was 188% of that in patients with low hs-CRP levels [RR = 1.88, 95%CI (1.41 ~ 2.52); *p* < 0.001; Figure 3A].

Eight studies (15, 19, 24, 25, 27, 28, 35, 38) involving 15,826 patients showed an association between per unit increase in the level of hs-CRP and the risk of recurrent stroke. Heterogeneity among studies was significant (*I*^2^ = 74.6%; *p* < 0.01), and thus a random-effects model was used. Meta-analysis showed that the risk of recurrent stroke increased by 3% for each unit increase in hs-CRP levels [RR = 1.03, 95% CI (1.01 ~ 1.04); *p* = 0.003; Figure 3B].

#### 3.3.3. Relationship between hs-CRP levels and poor prognosis

**Figure 4:**
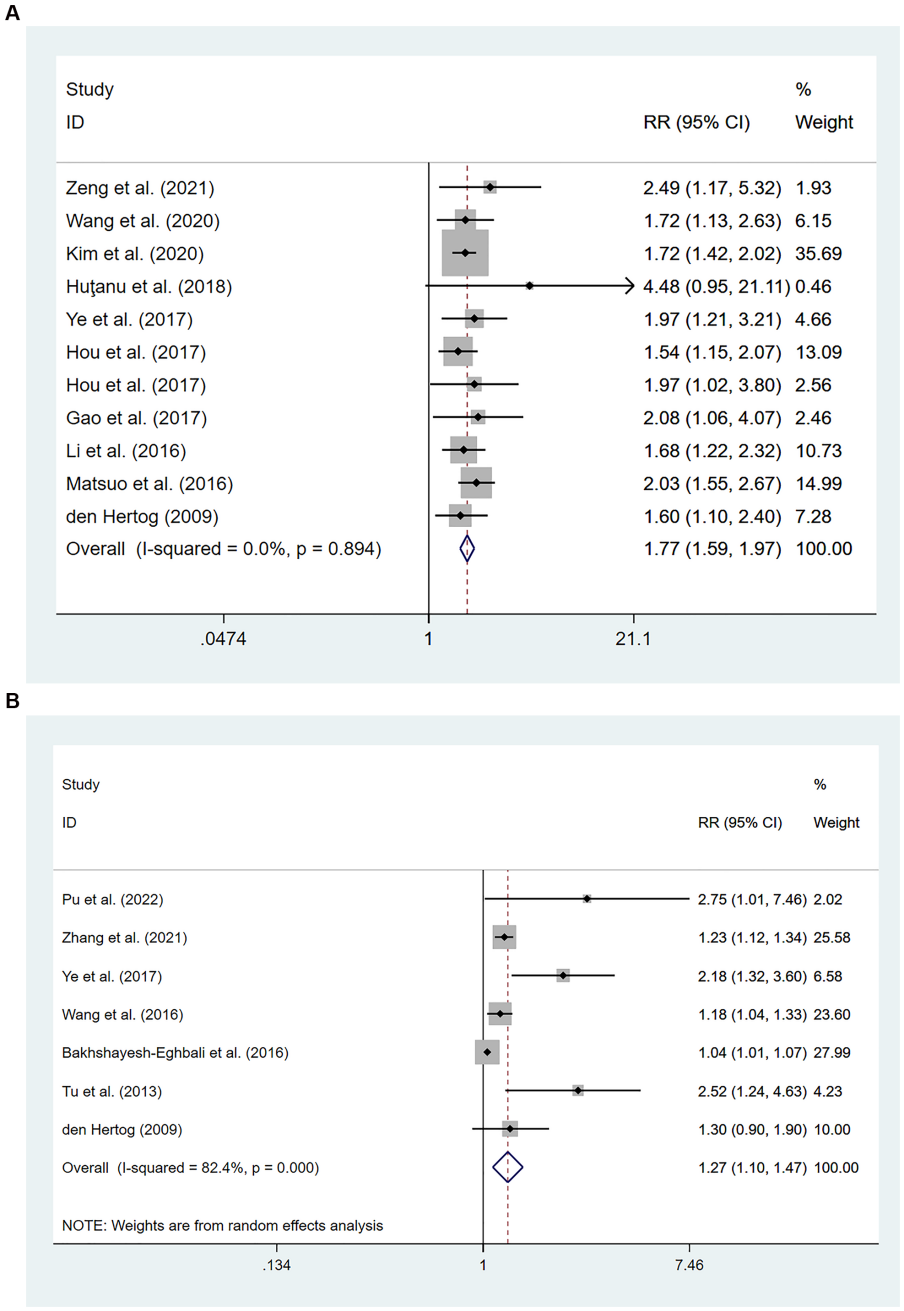
Forest plots showing risk ratios with 95% confidence intervals of poor outcome in ischemic stroke patients **(A)** the highest versus the lowest C-reactive protein level category; **(B)** per 1-SD rise in loge-hsCRP level.

Ten cohort studies involving a total of 11,184 patients (14, 20, 24, 29, 30, 33, 34, 41, 44, 46) evaluated the association between high levels of hs-CRP and poor prognosis in patients with AIS. Figure 4A showed that the incidence of poor prognosis in patients with high hs-CRP levels was 177% of that in patients with low hs-CRP levels [RR = 1.77, 95% CI (1.59 ~ 1.97); *p* < 0.001; Figure 4A]. There was no significant heterogeneity among these studies (*I*^2^ = 0%; *p* = 0.894).

Seven studies (31–33, 44, 45, 47, 48) with a total of 4,985 patients reported an association between per unit increase in the level of hs-CRP and poor prognosis. Meta-analysis showed that the risk of poor prognosis increased by 27% for each unit increases in hs-CRP levels [RR = 1.27, 95% CI (1.10 ~ 1.47); *p* = 0.001; Figure 4B], and there was significant heterogeneity among these studies (*I*^2^ = 82.4%; *p* < 0.01) (Figure 4).

### 3.4. Relationship between hs-CRP levels and all-cause mortality in patients with hemorrhagic stroke

**Figure 5:**
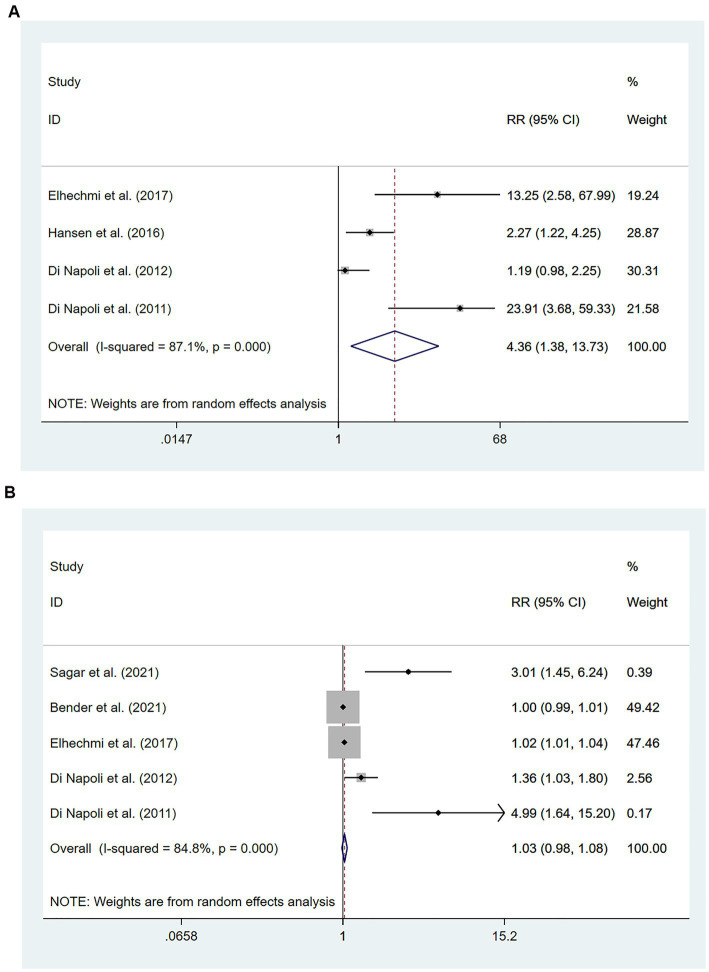
Forest plots showing risk ratios with 95% confidence intervals of mortality in hemorrhagic stroke patients **(A)** the highest versus the lowest C-reactive protein level category; **(B)** per 1-SD rise in loge-hsCRP level.

Participants with HS were included in six studies (49–54) involving 1,301 patients. Of these studies, four (51–54) involving 722 patients found that high hs-CRP levels at the time of admission were associated with an increased risk of all-cause mortality compared with low hs-CRP levels [RR = 4.36, 95% CI (1.38 ~ 13.73); *p* = 0.012; Figure 5A]. Significant heterogeneity was found between these studies (*I*^2^ = 87.1%; *p* < 0.01).

Five studies (49–51, 53, 54) involving 1,103 patients investigated the relationship between per unit increase in the level of hs-CRP and all-cause mortality. Meta-analysis revealed no statistical significance in the association between per unit increase in the level of hs-CRP and all-cause mortality among patients with HS [RR = 1.03, 95% CI (0.98 ~ 1.08); *p* = 0.238; Figure 5B]. Significant heterogeneity was observed between these studies (*I*^2^ = 84.8%; *p* < 0.01) (Figure 5).

### 3.5. Subgroup analysis

Subgroup analyses of patients with ischemic stroke were performed based on three outcome measures (Table 2). In the subgroup analysis where the level of hs-CRP was defined as a categorical variable, the correlation between high hs-CRP levels and mortality was found to be remarkable in models undergoing calibration [RR = 2.40, 95% CI (1.50 ~ 3.83); *p* < 0.001] or not [RR = 6.08, 95% CI (4.38 ~ 8.43); *p* < 0.001], with intragroup heterogeneity reduced (*I^2^* = 43.2%, *p* = 0.117; *I^2^* = 0.0%, *p* = 0.960). The subgroup analysis based on sample size showed that the correlation of hs-CRP levels with recurrent stroke and poor prognosis was not significantly affected by sample size. The correlation between hs-CRP and mortality was observed in the subgroup with a small sample size [RR = 4.23, 95% CI (2.54 ~ 7.04); *p* < 0.001]. Intragroup heterogeneity was significantly reduced regarding the risk of recurrent stroke (≥500: *I*^2^ = 0%, *p* = 0.561; <500: *I*^2^ = 0%, *p* = 0.603). Subgroup analysis based on the duration of follow-up also demonstrated that the correlation of hs-CRP levels with mortality and poor prognosis was not significantly affected by the duration of follow-up.

**Table 2:** Subgroup analysis for hs-CRP in AIS patients.

Subgroup	Number of studies	Pooled RRs (95% CI)	*p*-value	Heterogeneity	
				*I*^2^ (%)	*p*-value
Mortality (Categorical analysis)					
1. Follow-up duration					
3 months	5	3.13(1.52–6.46)	0.002	61.7	0.033
≥1 year	5	4.58(2.47–8.51)	<0.001	75.1	0.003
2. Model calibration					
Yes	6	2.40(1.50–3.83)	<0.001	43.2	0.117
No	4	6.08(4.38–8.43)	<0.001	0.0	0.960
3. Study size					
<500	8	4.23(2.54–7.04)	<0.001	69.3	0.002
≥500	2	2.72(0.78–9.52)	0.117	62.0	<0.001
Mortality (Continuous analysis)					
1. Follow-up duration					
3 months	4	1.58(1.37–1.83)	<0.001	24.2	0.266
≥1 year	4	1.21(1.00–1.48)	0.056	93.1	<0.001
2. Study size					
<500	5	1.47(1.18–1.82)	0.001	84.2	<0.001
≥500	3	1.37(0.94–1.98)	0.097	91.9	<0.001
Recurrent stroke (Categorical analysis)					
1. Study size					
<500	2	4.67(2.90–7.53)	<0.001	0.0	0.603
≥500	4	1.50(1.30–1.74)	<0.001	0.0	0.516
Recurrent stroke (Continuous analysis)					
1. Follow-up duration					
3 months	2	1.06(0.99–1.14)	0.100	79.7	0.026
≥1 year	6	1.02(1.00–1.03)	0.055	70.6	0.001
2. Model calibration					
Yes	5	1.12(1.04–1.19)	0.001	64.5	0.010
No	3	1.01(1.00–1.02)	0.077	69.8	0.037
3. Study size					
<500	3	1.03(1.00–1.06)	0.078	81.9	0.004
≥500	5	1.03(1.00–1.06)	0.023	75.0	0.001
4. Region					
Asian	6	1.02(1.00–1.04)	0.002	80.4	<0.001
Non-Asian	2	1.03(1.01–1.05)	0.017	0.0	0.588
Poor outcome(Categorical analysis)					
1. Follow-up duration					
3 months	7	1.75(1.56–1.96)	<0.001	0.0	0.786
≥1 year	3	1.91(1.42–2.57)	<0.001	0.0	0.700
Poor outcome(Continuous analysis)					
1. Follow-up duration					
3 months	5	1.24(1.04–1.49)	0.017	89.2	<0.001
≥1 year	2	1.52(0.84–2.76)	0.163	81.6	0.020
2. Study size					
<500	4	1.22(1.00–1.49)	0.055	78.9	0.003
≥500	3	1.40(1.06–1.84)	0.018	59.0	0.087
3. Region					
Asian	5	1.40(1.15–1.70)	0.001	67.8	0.014
Non-Asian	2	1.07(0.92–1.24)	0.355	26.0	0.245

In the subgroup analysis where the level of hs-CRP was defined as a continuous variable, the fact that hs-CRP levels were associated with all-cause mortality 3 months: [RR = 1.58, 95% CI (1.37 ~ 1.83); *p* < 0.001] and poor prognosis 3 months: [RR = 1.24, 95% CI (1.04 ~ 1.49); *p* = 0.017] was observed only in the subgroup followed up for 3 months where small heterogeneity pertaining to mortality was found (*I^2^* = 24.2%; *p* = 0.266). Region-based subgroup analysis demonstrated that there was a poor correlation between hs-CRP levels and recurrent stroke whether in the subgroup of Asia [RR = 1.02, 95% CI (1.00 ~ 1.04); *p* = 0.017] and the subgroup of non-Asian regions [RR = 1.03, 95% CI (1.01 ~ 1.05), *p* = 0.002]. The heterogeneity regarding recurrent stroke and poor prognosis was significantly reduced in the non-Asian subgroup (*I*^2^ = 0.00%, *p* = 0.588; *I^2^* = 26.0%, *p* = 0.245).

### 3.6. Sensitivity analysis and publication bias

Sensitivity analysis was conducted regarding all outcome measures investigated in the present study, and no significant changes were found in analysis results after removing included studies one by one, indicating good stability of results (Supplementary Figures S1–S3).

**Figure 6:**
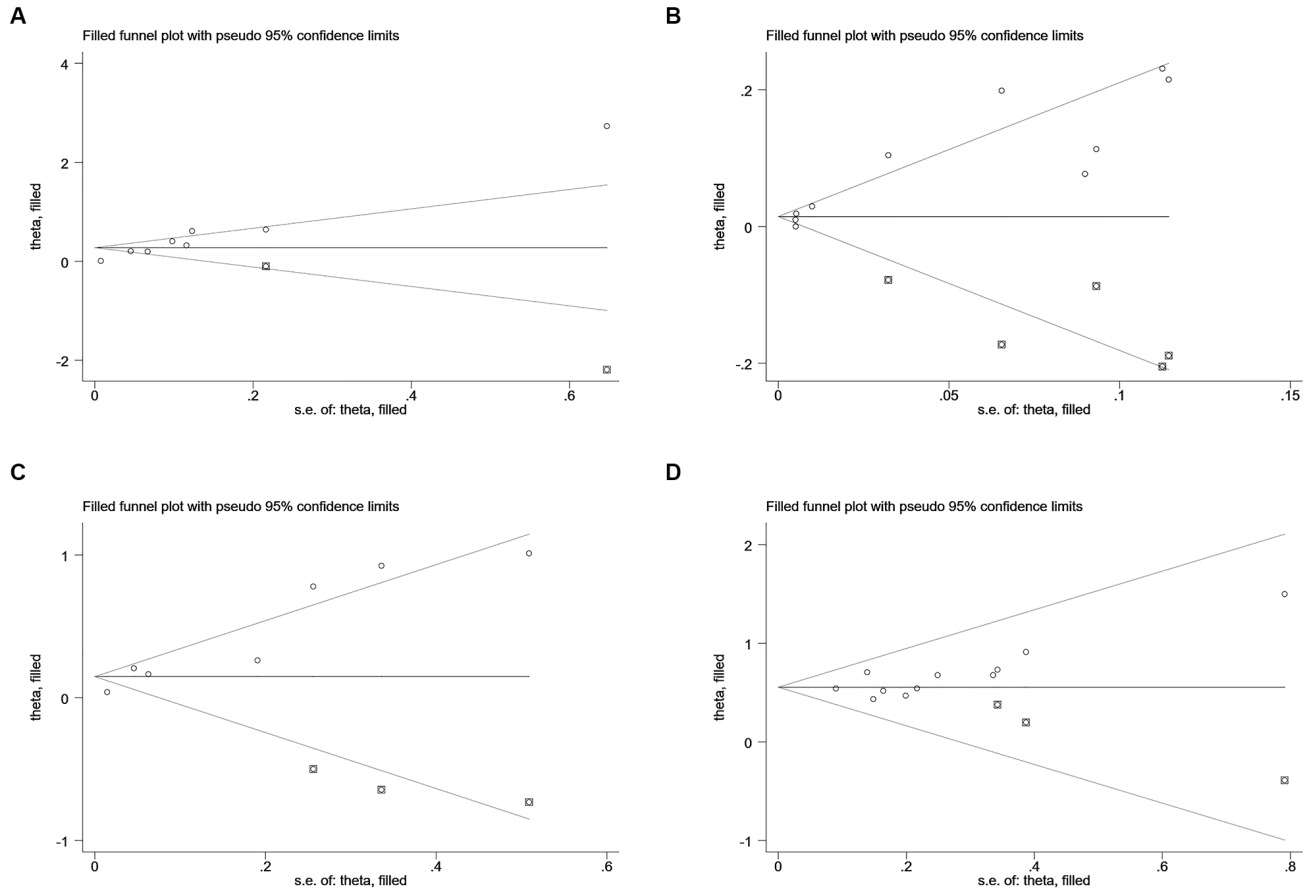
Funnel plots of **(A)** hsCRP (defined as per 1-SD increment) and mortality, **(B)** hsCRP (defined as per 1-SD increment) and recurrent stroke, **(C)** hsCRP (defined as per 1-SD increment) and poor outcome, and **(D)** hsCRP (defined as the highest versus the lowest) and poor outcome in ischemic stroke.

**Figure 7:**
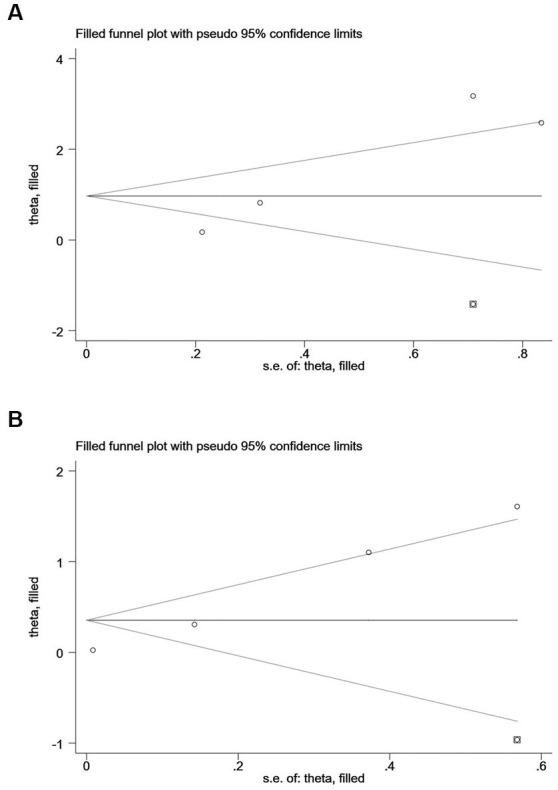
Funnel plots of **(A)** hsCRP (defined as the highest versus the lowest) and mortality, **(B)** hsCRP (defined as per 1-SD increment) and mortality in hemorrhagic stroke.

According to Begg’s and Egger’s test, publication bias was found across studies (*p* < 0.05, Supplementary Figures S6–S11) except for studies investigating mortality and the risk of recurrent stroke (*p* ≥ 0.05, Supplementary Figures S3–S5). After the trim-and-fill method was employed to adjust for publication bias (Figures 6–7), no statistical significance existed regarding the correlation of per unit increase in the level of hs-CRP with the risk of recurrent stroke [RR = 1.05, 95% CI (0.995 ~ 1.035); *p* = 0.147; Figure 6B] and poor prognosis [RR = 1.160, 95% CI (0.998 ~ 1.384); *p* = 0.053; Figure 6C] among patients with IS. Similar results were noted in the analysis on the correlation of hs-CRP levels and mortality among patients with HS [RR = 2.64, 95% CI (0.874 ~ 7.978); *p* = 0.085; Figure 7A; RR = 1.43,95% CI (0.919 ~ 2.210), *p* < 0.001; Figure 7B], suggesting that publication bias did not affect our findings.

## 4. Discussion

The meta-analysis of 39 studies contributed to a comprehensive investigation for the first time into the correlation of hs-CRP levels with mortality, recurrent stroke, and poor prognosis after stroke. Compared to patients with IS who had low hs-CRP levels upon admission, those with high hs-CRP levels were more prone to death, recurrent stroke, and poor prognosis. The same trend was noted in patients with HS, given their mortality. Per unit increase in the level of hs-CRP was associated with mortality, recurrent stroke, and poor prognosis among patients with IS, while it was not related to mortality among patients with HS.

In this study, the prognostic ability of hs-CRP levels in patients with IS was confirmed. However, results contrary to those in previous studies were obtained in the analysis of continuous variables regarding HS and mortality (49). Among the included studies, there was a significant difference between the inpatient mortality investigated by Bender et al. (50) and the duration of the follow-up (≥1 month) investigated by other included studies. The removal of the study by Bender et al. (50) produced results consistent with previous studies [RR = 1.65, 95% CI (1.05 ~ 2.59); *p* = 0.03; Supplementary Figure S12]. In addition, longitudinal analyses of different clinical outcomes were performed in the present study. A strong correlation of hs-CRP levels with outcome measures was found only when it came to mortality among patients with ischemic or hemorrhagic stroke [RR = 3.84, 95% CI (2.41 ~ 6.11), *p* < 0.001]; [RR = 4.36, 95% CI (1.38 ~ 13.73), *p* = 0.012], suggesting that the level of hs-CRP as a prognostic indicator of mortality may have greater clinical significance than that of recurrent stroke and poor prognosis. Evidence has shown that early recurrence after stroke is an independent factor for increased risk of death (55). By increasing the risk of recurrent stroke (19, 39), elevated hs-CRP levels are associated with the death of patients after stroke (56), strengthening the significance of hs-CRP levels in predicting mortality.

The activation process of the immune response for ischemic or hemorrhagic stroke is similar to the inflammatory response (57). After a stroke, neuronal cells die or are damaged, releasing damage-associated molecular patterns (DAMPs) that trigger local inflammation in the damaged brain area. The release of inflammatory factors increases the permeability of the blood–brain barrier (BBB), causing infiltration of peripheral immune cells into the lesion, which induces an inflammatory cascade and causes secondary brain injury (58). Inflammation is a key driving force in the development of atherosclerosis (59), which leads to stroke through a variety of mechanisms including plaque rupture, thrombosis, embolism, and hemodynamic impairment (6). Currently, there is ample evidence to support that reducing hs-CRP levels through anti-inflammatory interventions can improve stroke prognosis. Canakinumab (60), an anti-inflammatory drug, has been shown to significantly reduce the risk of recurrent cardiovascular events, including stroke, in patients with a history of myocardial infarction and elevated levels of hs-CRP. Statins have also been found to improve clinical prognosis in the same manner (61). In conclusion, there is a piece of clear evidence that anti-inflammatory interventions may improve the prognosis of stroke patients. However, further investigations are needed to elucidate the underlying mechanisms, which have significant implications for the specific treatment of stroke. Our study differs from previous research (16) in that blood samples were collected after stroke onset but before treatment initiation, reflecting mainly the impact of acute-phase pro-inflammatory cytokines on prognosis. This provides additional evidence for the important role of anti-inflammatory therapy in the acute phase of stroke.

This meta-analysis revealed the correlation between hs-CRP levels and the prognosis of patients with stroke, but results should be interpreted with caution, given the great heterogeneity across included studies with no single study found in the sensitivity analysis to reduce the heterogeneity. Subgroup analyses were performed to disclose the source of heterogeneity. The heterogeneity in view of the mortality among patients with IS in the models undergoing calibration or not was reduced, respectively, (*I^2^* = 43.2%, *p* = 0.117; *I*^2^ = 0.0%, *p* = 0.960), possibly indicating that model calibration contributed to the heterogeneity regarding mortality. When the level of hs-CRP was defined as a continuous variable, small heterogeneity pertaining to mortality was only observed within the subgroup of 3-month follow-up (*I^2^* = 24.2%; *p* = 0.266), which may be related to the difference in follow-up duration between studies lasting for over 1 year. In addition, the sample size may be a potential source of heterogeneity regarding recurrent stroke (≥500: *I*^2^ = 0%, *p* = 0.561; < 500: *I*^2^ = 0%, *p* = 0.603). However, the heterogeneity in poor prognosis was only significantly reduced within the non-Asian subgroup (*I*^2^ = 26.0%, *p* = 0.245), which may be related to regional publication preferences. There may be a certain correlation between prognosis and stroke subtypes, but this was not investigated in the present study due to insufficient data, emphasizing the need for additional research.

There are some limitations to this study. First, factors that were adjusted such as autoimmune diseases or chronic inflammatory diseases among participants during the research process were not realized across included studies, which may be an important source of heterogeneity across studies. Second, per unit increase in hs-CRP levels was found to not be associated with poor prognosis and recurrent stroke after the trim-and-fill method was applied, calling for negative results to be reported in future research to avoid overestimating the clinical significance of hs-CRP. Third, the general development trend instead of the panorama of inflammation is reflected by hs-CRP levels due to complicated inflammation mechanisms, which is coupled with a single measurement of hs-CRP levels performed in each included study. Therefore, whether the dynamic changes in hs-CRP levels can provide additional prognostic significance remains to be determined.

Taken together, higher hs-CRP levels upon admission are associated with poor prognosis after stroke, including ischemic and hemorrhagic stroke. Elevated hs-CRP levels may further increase the recurrence and mortality of cerebral infarction or cerebral hemorrhage in patients with stroke. Moreover, the level of hs-CRP upon admission is a good prognostic biomarker for patients with stroke during the follow-up of 3 months.

## Data availability statement

The original contributions presented in the study are included in the article/Supplementary material, further inquiries can be directed to the corresponding author.

## Author contributions

BH and LC: conceptualization. LC: methodology and writing original draft preparation. LC, MW, CY, and YW: formal analysis and investigation. BH: writing review and editing, funding acquisition, resources, and supervision. LC, MW, CY, YW, and BH commented on previous versions of the manuscript. All authors contributed to the article and approved the submitted version.

## Funding

This work was supported by the National Natural Science Foundation of China (No. 82104762).

## Conflict of interest

The authors declare that the research was conducted in the absence of any commercial or financial relationships that could be construed as a potential conflict of interest.

## Publisher’s note

All claims expressed in this article are solely those of the authors and do not necessarily represent those of their affiliated organizations, or those of the publisher, the editors and the reviewers. Any product that may be evaluated in this article, or claim that may be made by its manufacturer, is not guaranteed or endorsed by the publisher.
