# Supplementary material for: The role of high-sensitivity C-reactive protein serum levels in the prognosis for patients with stroke: a meta-analysis
Source: Front Neurol. 2023 Jun 5;14:1199814. doi: 10.3389/fneur.2023.1199814 (PMC10278886; doi:10.3389/fneur.2023.1199814)
Supplement: Supplementary file 1 [file Data_Sheet_1.docx]

**Supplemental methods**

**Search Terms used for Web of Science 28^th^** **October 2022**

#1 (((((((((((((TI=(Stroke)) OR TI=(intracerebral haemorrhage)) OR TI=(TIA)) OR TI=(Transient ischemic attack)) OR TI=(Cerebrovascular disease*)) OR TI=(Strokes)) OR TI=(Cerebrovascular Accident)) OR TI=(Cerebrovascular Accidents)) OR TI=(CVA )) OR TI=(CVAs )) OR TI=(Cerebrovascular Apoplexy)) OR TI=(Brain Vascular Accident)) OR TI=(Brain Vascular Accidents)) OR TI=(Apoplexy)

#2 (((TS=(C-reactive protein)) OR TS=(high-sensitivity CRP)) OR TS=(hs-CRP)) OR TS=(hsCRP)

#3 ((((((TS=(observational)) OR TS=(cohort)) OR TS=(case control)) OR TS=(cross sectional)) OR TS=(Follow up)) OR TS=(prospective)) OR TS=(retrospective)

#4 #1 AND #2 AND #3

**Search Terms used for Embase 28^th^ October 2022**

#1 'cerebrovascular accident'/exp

#2 stroke:ti OR 'intracerebral haemorrhage':ti OR tia:ti OR 'transient ischemic attack':ti OR 'cerebrovascular disease*':ti OR strokes:ti OR 'cerebrovascular accident':ti OR 'cerebrovascular accidents':ti OR cva:ti OR cvas:ti OR 'cerebrovascular apoplexy':ti OR 'brain vascular accident':ti OR 'brain vascular accidents':ti OR apoplexy:ti

#3 #1 OR #2

#4 'c reactive protein'/exp

#5 'c-reactive protein':ab,ti OR 'high-sensitivity crp':ab,ti OR 'hs crp':ab,ti OR hscrp:ab,ti

#6 #4 OR #5

#7 observational OR cohort OR (case AND control) OR (cross AND sectional) OR (follow AND up) OR prospective OR retrospective

#7 observational OR cohort OR (case AND control) OR (cross AND sectional) OR (follow AND up) OR prospective OR retrospective

**Search Terms used for Pubmed 28^th^ October 2022**

1. "Stroke"[Mesh]
2. (((((((((((((Stroke[Title/Abstract]) OR (intracerebral haemorrhage[Title/Abstract])) OR (TIA[Title/Abstract])) OR (Transient ischemic attack[Title/Abstract])) OR (Cerebrovascular disease*[Title/Abstract])) OR (Strokes[Title/Abstract])) OR (Cerebrovascular Accident[Title/Abstract])) OR (Cerebrovascular Accidents[Title/Abstract])) OR (CVA[Title/Abstract])) OR (CVAs[Title/Abstract])) OR (Cerebrovascular Apoplexy[Title/Abstract])) OR (Brain Vascular Accident[Title/Abstract])) OR (Brain Vascular Accidents[Title/Abstract])) OR (Apoplexy[Title/Abstract])
3. ("Stroke"[Mesh]) OR ((((((((((((((Stroke[Title/Abstract]) OR (intracerebral haemorrhage[Title/Abstract])) OR (TIA[Title/Abstract])) OR (Transient ischemic attack[Title/Abstract])) OR (Cerebrovascular disease*[Title/Abstract])) OR (Strokes[Title/Abstract])) OR (Cerebrovascular Accident[Title/Abstract])) OR (Cerebrovascular Accidents[Title/Abstract])) OR (CVA[Title/Abstract])) OR (CVAs[Title/Abstract])) OR (Cerebrovascular Apoplexy[Title/Abstract])) OR (Brain Vascular Accident[Title/Abstract])) OR (Brain Vascular Accidents[Title/Abstract])) OR (Apoplexy[Title/Abstract]))
4. "C-Reactive Protein"[Mesh]
5. (((C-reactive protein[Title/Abstract]) OR (high-sensitivity CRP[Title/Abstract])) OR (hs-CRP[Title/Abstract])) OR (hsCRP[Title/Abstract])
6. ("C-Reactive Protein"[Mesh]) OR ((((C-reactive protein[Title/Abstract]) OR (high-sensitivity CRP[Title/Abstract])) OR (hs-CRP[Title/Abstract])) OR (hsCRP[Title/Abstract]))
7. ((((((observational) OR (cohort)) OR (case control)) OR (cross sectional)) OR (Follow up)) OR (prospective)) OR (retrospective)
8. ((("Stroke"[Mesh]) OR ((((((((((((((Stroke[Title/Abstract]) OR (intracerebral haemorrhage[Title/Abstract])) OR (TIA[Title/Abstract])) OR (Transient ischemic attack[Title/Abstract])) OR (Cerebrovascular disease*[Title/Abstract])) OR (Strokes[Title/Abstract])) OR (Cerebrovascular Accident[Title/Abstract])) OR (Cerebrovascular Accidents[Title/Abstract])) OR (CVA[Title/Abstract])) OR (CVAs[Title/Abstract])) OR (Cerebrovascular Apoplexy[Title/Abstract])) OR (Brain Vascular Accident[Title/Abstract])) OR (Brain Vascular Accidents[Title/Abstract])) OR (Apoplexy[Title/Abstract]))) AND (("C-Reactive Protein"[Mesh]) OR ((((C-reactive protein[Title/Abstract]) OR (high-sensitivity CRP[Title/Abstract])) OR (hs-CRP[Title/Abstract])) OR (hsCRP[Title/Abstract])))) AND (((((((observational) OR (cohort)) OR (case control)) OR (cross sectional)) OR (Follow up)) OR (prospective)) OR (retrospective))

**Search Terms used for Cochrane Library 28^th^ October 2022**

#1 MeSH descriptor: [Stroke] explode all trees

#2 (Stroke):ti,ab,kw OR (intracerebral haemorrhage):ti,ab,kw OR (TIA):ti,ab,kw OR (Transient ischemic attack):ti,ab,kw OR (Cerebrovascular disease*):ti,ab,kw

#3 (Strokes):ti,ab,kw OR (Cerebrovascular Accident):ti,ab,kw OR (Cerebrovascular Accidents):ti,ab,kw OR (CVA):ti,ab,kw OR (CVAs):ti,ab,kw

#4 (Cerebrovascular Apoplexy):ti,ab,kw OR (Brain Vascular Accident):ti,ab,kw OR (Brain Vascular Accidents):ti,ab,kw OR (Apoplexy):ti,ab,kw

#5 #1 or #2 or #3 or #4

#6 MeSH descriptor: [C-Reactive Protein] explode all trees

#7 (C-reactive protein):ti,ab,kw OR (high-sensitivity CRP):ti,ab,kw OR (hs-CRP):ti,ab,kw OR (hsCRP):ti,ab,kw

#8 #6 or #7

#9 (observational) OR (cohort) OR (case control) OR (cross sectional) OR (Follow up)

#10 (prospective) OR (retrospective)

#11 #9 or #10

#12 #5 and #8 and #11
